# Differential expression level of cytokeratin 8 in cells of the bovine nucleus pulposus complicates the search for specific intervertebral disc cell markers

**DOI:** 10.1186/ar2931

**Published:** 2010-02-12

**Authors:** Audrey Gilson, Mathias Dreger, Jill PG Urban

**Affiliations:** 1 Department of Physiology, Anatomy and Genetics, University of Oxford, South Parks Road, Oxford, OX1 3QX, UK; 2 Caprotec Bioanalytics GmbH, Volmerstrasse 5, Berlin 12489, Germany

## Abstract

**Introduction:**

Development of cell therapies for repairing the intervertebral disc is limited by the lack of a source of healthy human disc cells. Stem cells, particularly mesenchymal stem cells, are seen as a potential source but differentiation strategies are limited by the lack of specific markers that can distinguish disc cells from articular chondrocytes.

**Methods:**

We searched for markers using the differential in-gel electrophoresis proteomic technology to compare proteins of bovine nucleus pulposus cells, phenotypically similar to mature human nucleus cells, with those of bovine articular chondrocytes. In the cohort of the differentially expressed proteins identified by mass spectrometry, cytokeratin 8 (CK8) was further validated by immunostaining of freshly isolated cells and frozen tissue sections using monoclonal antibodies.

**Results:**

We identified a set of 14 differentially expressed proteins. Immunohistochemistry showed that only a subset of cells (approximately 10%) was positive for one of these proteins, CK8, an intermediate filament protein present in epithelial but not mesenchymal cells. In tissue sections, CK8-positive cells were seen in all discs examined and appeared as small isolated clusters surrounded by gelatinous matrix. Notochordal nucleus pulposus cells from pig, phenotypically similar to human infant nucleus pulposus cells, were all CK8-positive. The mesenchymal intermediate filament protein vimentin was present in all bovine and porcine nucleus pulposus cells.

**Conclusions:**

The notochordal cell population is reported to disappear from the nucleus pulposus of bovine discs before birth and from human discs in childhood. However our finding of the co-expression of vimentin and CK8 in small isolated clusters of the bovine nucleus pulposus cells indicates that a subpopulation of notochordal-like cells remains in the mature bovine disc. This finding agrees with reports in the literature on co-expression of cytokeratins and vimentin in adult human discs. As notochordal cells produce factors that promote matrix production, the CK8-positive subpopulation could have important implications for activity and survival of the nucleus pulposus, and should be considered in development of cell therapies for disc repair. In addition, the finding of differential expression of proteins in the cell population of nucleus pulposus has implications with regard to the search for specific markers.

## Introduction

Low back pain constitutes a major health problem and a huge economic burden [1]. It is highly associated with degeneration of the intervertebral disc [2]. The earliest degenerative changes are seen in the central region of the disc, the nucleus pulposus (NP) [3], and are characterized initially by loss of proteoglycans and finally by loss of matrix integrity [3,4]. Treatments on offer are still mainly palliative or surgical and do not improve the ability of the disc to regain its original architecture and function. Biological approaches, particularly those that aim to produce a tissue-engineered disc or to insert cells into the damaged NP to regenerate the matrix and restore the disc's biomechanical function, are seen as a potential alternative [5].

Implementation of cell therapies for repairing the disc is limited by lack of an appropriate cell source as healthy disc cells are not available for expansion and treatment [6]. Efforts therefore have concentrated on differentiating stem cells, particularly mesenchymal stem cells (MSCs), into disc cells both *in vitro *and *in vivo *[7]. The success of the differentiation protocols used is, however, uncertain as the markers mainly used (such as expression of collagen II, aggrecan, and sox 9 [8]) are expressed by all cartilage cells. The MSCs thus could be differentiating equally well into articular chondrocytes (ACs) as into 'disc-like cells' [8,9]. It is, however, vital for successful repair that a matrix that is permissive of the disc's biomechanical requirements be produced by the differentiated cells. Although disc cells and ACs express many of the same macromolecules, there are distinct differences in the overall composition and biomechanical properties of the matrix produced. Disc nucleus cells, for instance, produce a loose collagen II network that supports the disc's requirements for flexibility while ACs produce a much more rigid matrix through a tightly cross-linked collagen II network; these differences possibly arise from differences in splice variants and post-translational modifications of the collagen molecules produced by these two different cell types [10-12]. Specific disc cell markers to ensure that MSCs differentiate into disc cells rather than into some other cartilaginous cell type are thus an essential requirement for success of cell implantation therapies.

Microarray screens have been used to define markers that will distinguish disc cells from other cartilage cells [13-15]. In addition, expression of HIF (hypoxia-inducible factor) and GLUT (glucose transporter) isoforms have been suggested as markers [13,16]. In general, these studies, while identifying differences in the level of expression of a number of genes or proteins between annulus cells, nucleus cells, and ACs, have found no specific markers, apart from CD24. Moreover, the studies have been carried out mainly on rats, which because of a difference in NP cell phenotype are not a good model for the human disc nucleus [17]. The NP of all mammals, including humans, originates from the notochord [18,19] and in early fetal life contains clusters of large vacuolated cells producing a fluid matrix of low collagen content [20,21]. In some animals such as rats, pigs, and rabbits, these notochordal cells persist well into adulthood and even throughout life [17,22]. However, in other species, including humans and cattle, the notochordal cell clusters disappear early in life and are replaced by the smaller cells of chondrocyte-like appearance seen in the adult NP; these produce a firmer, more collagenous matrix [20-23]. Hence, as shown in a recent study [24], the markers produced from studies on rodents may not be specific for human discs and may not be relevant for repair studies on human discs.

Here, we describe another approach to the search for specific disc cell markers. First, as non-degenerate adult human discs are not available to us, we used cells from the nucleus of young adult bovine caudal discs; these cells are thought be phenotypically similar to adult human nucleus cells and produce a matrix similar to that of adult humans [17,25]. Second, we looked for markers at the protein rather than at the gene expression level. We used the differential in-gel electrophoresis (DIGE) technology to compare disc cells with ACs as these two cell types have a similar morphology and both produce typical cartilaginous markers such as aggrecan and collagen II. Indeed, disc nucleus cells are often referred to as chondrocyte-like cells [26]. We found 14 proteins that were expressed only by disc or only by cartilage cells. Here, we concentrate on findings related to cytokeratin 8 (CK8), an intermediate filament protein strongly expressed by our disc cell preparation and not expressed at the protein level by ACs or cells from the annulus fibrosus (AF). We found that CK8 was differentially expressed in cells of the bovine NP. The apparent cellular heterogeneity raises questions about the search for specific cellular markers to identify cells of the mature NP.

## Materials and methods

### Tissue harvest and cell isolation

Caudal intervertebral discs from 18- to 24-month-old bovine steers (non-notochordal discs) and 6-month-old pigs (notochordal discs) and bovine metacarpal phalangeal joints were obtained from the local abattoir and dissected under aseptic conditions within 6 hours after slaughter. In all, for DIGE analysis, six independent (that is, different animals) isolations of bovine NP cells and six independent isolations of ACs were carried out. In addition, for immunostaining and cell measurement, we carried out six more independent cell isolations from bovine discs, six from pig discs, and four from bovine articular cartilage. Bovine NP cells, AF cells, and ACs were isolated by an overnight enzymatic digestion using 0.05% (wt/vol) and 0.075% (wt/vol) type I collagenase (Sigma-Aldrich, Dorset, UK), respectively, in serum-free Dulbecco's modified Eagle's medium (DMEM) (catalog no. 22320; Invitrogen Corporation, Paisley, UK) containing antibiotics and antimycotics (Invitrogen Corporation) as previously reported [27]. Likewise, notochordal cells were obtained from the pig NP by a 1-hour digestion in 0.025% (wt/vol) protease (type XIV; Sigma-Aldrich) serum-free DMEM followed by an overnight digestion with 0.0125% (wt/vol) type I collagenase in DMEM supplemented with 10% (vol/vol) fetal bovine serum (Invitrogen Corporation) in accordance with the protocol described by Guehring and colleagues [28]. After digestion at 37°C, the cells were filtered through an 80-μm pore mesh and washed. A 1-hour treatment with a non-enzymatic cell dissociation solution (Sigma-Aldrich) was used in order to dissociate and count the notochordal cells and assess for viability using trypan blue exclusion.

### Total protein extraction and sample preparation for two-dimensional gel analysis

Proteins were extracted from freshly isolated cells using RIPA lysis buffer (1% NP-40, 0.5% sodium deoxycholate, 0.1% SDS, 2 mM PMSF (phenylmethylsulphonyl fluoride), 1 mM sodium orthovanadate, 1x protease inhibitor cocktail) (catalog no. SC-24948; Santa Cruz Biotechnology, Inc., Santa Cruz, CA, USA) in accordance with the manufacturer's instructions. Proteins in the supernatant were concentrated by chloroform/methanol precipitation. The resulting pellet was resuspended in sample buffer composed of 2 M thiourea, 7 M urea, and 4% CHAPS (Sigma-Aldrich) (pH 8.5) to a protein concentration of 5 mg/mL, and pH was adjusted to pH 8.0. Protein concentrations were determined by spectrophotometry using the DC Bio-Rad assay (Bio-Rad Laboratories, Hemel Hempstead, UK) with bovine serum albumin as a standard.

### Two-dimensional differential in-gel electrophoresis of freshly isolated cells

Fifty micrograms of total protein from freshly isolated bovine NP cells and freshly isolated ACs were respectively labelled with 400 pmol of Cy3 or Cy5 fluorescent dye (GE Healthcare, Little Chalfont, UK), mixed, and adjusted with isoelectric focusing (IEF) rehydration buffer composed of 2 M thiourea, 7 M urea, 4% CHAPS, 0.5% immobilized pH gradient (IPG) buffer (GE Healthcare), and 100 mM DTT (dithiothreitol) (Sigma-Aldrich) to obtain a final volume of 350 μL. The protein sample was allowed to rehydrate an 18-cm IPG dry strip (IPG 3-10 NL; GE Healthcare) for 12 hours followed by IEF in an Ettan IPGphor (GE Healthcare) (250 V fixed for 1 hour, 500 V fixed for 1 hour, 1,000 V fixed for 1 hour, 1,000 to 8,000 V gradient for 2 hours, and 10,000 V fixed for 1 hour). The gel strip was then applied onto the top of a 10% poly-acrylamide gel to enable the separation of the proteins according to their molecular weight (10 mA/gel for 1 hour and then 250 V limit for 4 hours). After two-dimensional (2D) PAGE, the gels were scanned on a Typhoon 9400 scanner and spots were visualized using DeCyder version 5.1.2 imaging software (both GE Healthcare).

### Peptide mass fingerprinting by mass spectrometry

Protein spots were excised manually from a colloidal Coomassie brilliant Blue G-250 (Bio-Rad Laboratories) preparative gel containing 100 μg of protein extracted from freshly isolated NP cells. The samples were digested according to standard procedures using proteomics-grade trypsin (Sigma-Aldrich) and further desalted using C-18 tips (ZipTip; Millipore, Chandlers Ford, UK). The digests were then spotted onto a matrix-assisted laser desorption/ionization (MALDI) target plate with α-cyano-4-hydroxycinnamic acid (Sigma-Aldrich) as the matrix. The monoisotopic peptide mass fingerprinting spectra obtained by the MALDI TOF/TOF (tandem time-of-flight) mass spectrometer (Bruker Ultraflex; Bruker Daltonics, Coventry, UK) were matched against the non-human, non-rodent sequences of the non-redundant NCBI (National Center for Biotechnology Information) database using the MASCOT (Matrix Science, London, UK) search engine. The following searching criteria were used to identify proteins: peptide mass accuracy of 50 ppm, one missed trypsin cleavage, carboxamidomethylation of cysteine residues, and oxydation of methionine residue.

### Immunofluorescence staining on cells and frozen tissue sections

Isolated cells (bovine NP, AF, AC, or pig notochordal) were settled onto coverslips and were fixed in methanol at -20°C for 30 minutes unless otherwise stated. Strips of bovine NP and pig notochordal discs, 5 to 10 mm in thickness, were snap-frozen and cut transversally with a cryostat microtome. The 25-μm frozen tissue sections obtained were fixed in a similar way to the isolated cells. After blocking in 1% bovine serum albumin-phosphate-buffered saline (PBS), the samples were further incubated in the presence of a monoclonal antibody to CK8 (5 μg/mL) (catalog no. SM3079P; Acris Antibodies GmbH, Hiddenhausen, Germany) either for 1 hour at room temperature or overnight at 4°C. For double-staining, simultaneous incubation with monoclonal antibodies raised against vimentin of IgM isotype (catalog no. V2258; Sigma-Aldrich) and CK8 of IgG1 isotype was performed for 1 hour at room temperature at concentrations of 25 and 5 μg/mL, respectively. Samples were then incubated with a fluorescein isothiocyanate-conjugated secondary antibody (Dako, Ely, UK) or a mix of 488 fluor-conjugated anti-IgM (Invitrogen Corporation) and 594 fluor-conjugated anti-IgG1 (Invitrogen Corporation) for 1 hour in the dark at room temperature. Following a wash with PBS, the samples were mounted on glass slides using Vectashield medium containing DAPI (4'-6-diamidino-2-phenylindole) (Vector Laboratories Inc., Peterborough, UK) for DNA counterstain. Slides were visualized using a Leica microscope (Leica, Wetzlar, Germany). Immunofluorescence images were converted into an mrc file and subsequently imported into the IMOD (Image MODeling) package (University of Colorado, Boulder, CO, USA) [29]. For measurement of cell size, a fraction aliquot of the cell suspension isolated from one animal was fixed and stained as described above; the cells were individually contoured (n = 500) and surface measurements were then calculated by the software.

### Toluidine blue staining of vertebral body growth plates

The vertebral bodies adjacent to the uppermost disc analyzed for the presence of CK8-positive cells were collected, cut mid-sagitally, and incubated with freshly prepared 1% toluidine blue solution (Sigma-Aldrich). This cationic dye, known to stain sulphated-glycosaminoglycans, was used to identify growth plate cartilage of the vertebral bodies; the absence of toluidine blue staining showed growth plate closure and skeletal maturity.

## Results

### Two-dimensional proteome map of freshly isolated bovine nucleus pulposus cells

**Figure 1:**
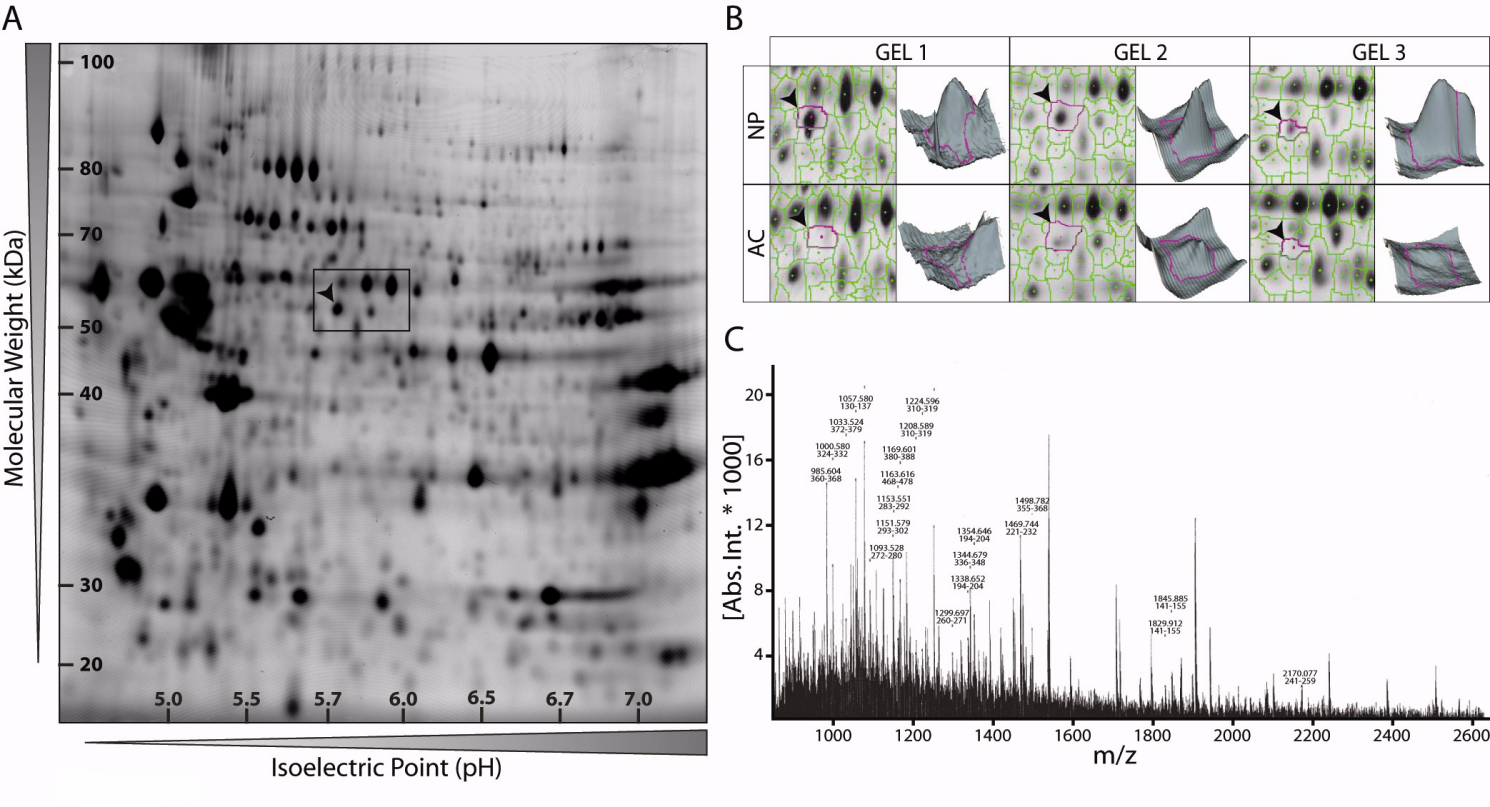
**Proteome map of freshly isolated bovine nucleus pulposus (NP) cells and identification of cytokeratin 8 (CK8) by mass spectrometry**. **(a) **Representative two-dimensional CyDye3-stained gel of total NP cell proteins separated according to their isoelectric point and molecular weight and **(b) **analysis of the NP and articular chondrocyte (AC) spot patterns by DeCyder software. **(c) **Mass spectrum of the spot indicated by an arrowhead in (a) and (b) identified by peptide mass fingerprinting as CK8.

To identify specific markers of freshly isolated bovine NP cells, the Cy3- and Cy5-labelled water-soluble protein fractions of NP cells and ACs were resolved by 2D gel electrophoresis. In total, six analytical 2D gels corresponding to independent cell isolations were analyzed using the DeCyder software, which was initially set up to detect an estimated 2,500 protein spots on a single gel (Figure 1a, representative 2D spot pattern of NP Cy3-labelled protein sample). A corresponding enlarged view of the gel area boxed in Figure 1a has been depicted for the two different cell types (NP and AC) on three different gels to emphasize the high level of protein resolution and to demonstrate the reproducibility of the 2D profiles where the spot indicated by an arrowhead is consistently present in the NP fraction and absent in the AC fraction (Figure 1b). Individual gel spot intensity was converted into a volumetric map for a three-dimensional visualization of the spot pattern (Figure 1b). The relatively abundant spot indicated by an arrowhead in Figure 1a and 1b was excised manually from a colloidal Coomassie Blue-stained preparative gel and digested with trypsin. The resulting peptide mixture was subjected to peptide mass fingerprinting analysis to generate a mass spectrum (Figure 1c) with experimentally measured peptide masses. The protein was identified as CK8 based on 22 peptide matches, 42% sequence coverage, a Mascot score of 107, and molecular mass and pI values of 55 kDa and 5.7, respectively. Similarly, 13 other proteins found either only in nucleus or only in cartilage cells were identified. These differences arose mainly from post-translational modifications of metabolic enzymes (the study of which will be the subject of a separate publication, which is in preparation), and this is possibly why they were not identified on mRNA microarrays [14,15].

### Validation of cytokeratin 8 expression in freshly isolated bovine nucleus pulposus cells

**Figure 2:**
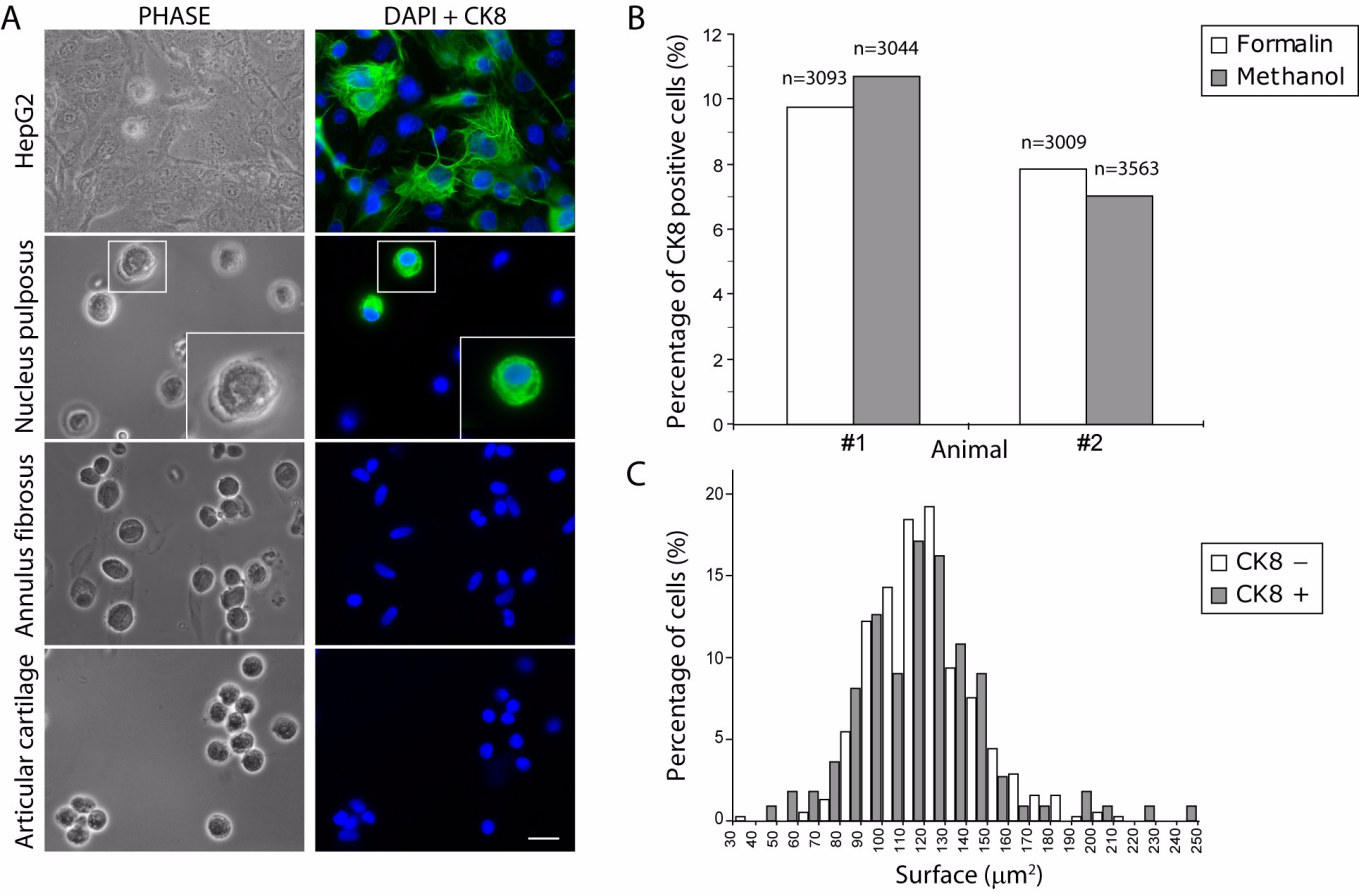
**A subpopulation of bovine nucleus pulposus (NP)-derived cells stains positive for cytokeratin 8 (CK8)**. **(a) **Immunofluorescence staining of CK8 performed on HepG2 cells, used here as a positive control, and on freshly isolated NP cells, annulus fibrosus cells, and articular cartilage cells. Cells were also imaged under classical phase-contrast microscopy (PHASE), and nuclei were visualized with DAPI (4'-6-diamidino-2-phenylindole) (scale bar = 20 μm). Note that some of the NP CK8-positive cells were single cells of appearance similar to the CK8-negative cells shown here, whereas others were in clusters of two or more cells (not shown). Insets show the boxed NP CK8-positive cell at a higher magnification. **(b) **Histogram of CK8-positive cell counts in formalin-fixed and in methanol-fixed freshly isolated NP cells. **(c) **Size distribution of CK8-positive and -negative cells on a surface area basis, analyzed using IMOD (Image MODeling) software.

We concentrated on this cytoskeletal protein because CK8 was highly expressed by disc cells, because cytokeratins are expressed by notochordal cells including those of the human NP [30], and because they have been identified in microarrays of rat and dog discs [14,15]. To confirm expression in freshly isolated cells, indirect immunostaining was performed using a monoclonal antibody with the reactivity of the antibody tested by using HepG2 cells as a positive control (Figure 2a). Surprisingly, in view of the CK8 expression level, we found that only a subset of the cells was immunopositive for CK8 (mean ± standard deviation: 11.61% ± 3.13%, counted on five individual animals using methanol fixation). In these cells, it appeared as a dense network throughout the entire cytoplasm (Figure 2a). Few of the CK8-expressing cells were found as isolated cells and were mostly seen as pairs or as relatively small clusters of cells. To investigate whether the stained cells possibly arose from contamination by cells from the surrounding tissue, we checked for the expression of CK8 in AF cells. No fluorescent signal could be detected in this cell type (Figure 2a). We addressed other potential methodological artifacts associated with the staining procedure by using formalin instead of methanol as a fixative and found that the number of CK8-positive cells was similar for both treatments (Figure 2b). Second, we verified the efficiency of the permeabilization step by treating NP cells with increasing concentrations of Igepal CA-630 (Sigma-Aldrich). The percentage of cells showing positive immunostaining with increasing concentrations of detergent did not change significantly (data not shown). Taken together, these data show that the CK8 cell population is strictly derived from the NP and that our staining procedure is optimal for the detection of this intermediate filament protein.

As notochordal cells are significantly larger in diameter than the chondrocyte-like cells from the mature NP and contain visible inclusions [20,28,31], we measured the diameter of CK8-positive and -negative cells from the bovine NP. We found no readily visible inclusions and no difference in average cell diameter (Figure 2c) between these two populations of cells; however, at the very tails of the distribution, 4.5% of the CK8-positive cells had a surface area 60% above the average compared with only 1% of the CK8-negative cells. Thus, apart from this small fraction of larger cells, the CK8-positive cells could not be identified on the basis of cell size or morphology.

### Expression of cytokeratin 8 in disc nucleus pulposus tissues from notochordal and non-notochordal species

**Figure 3:**
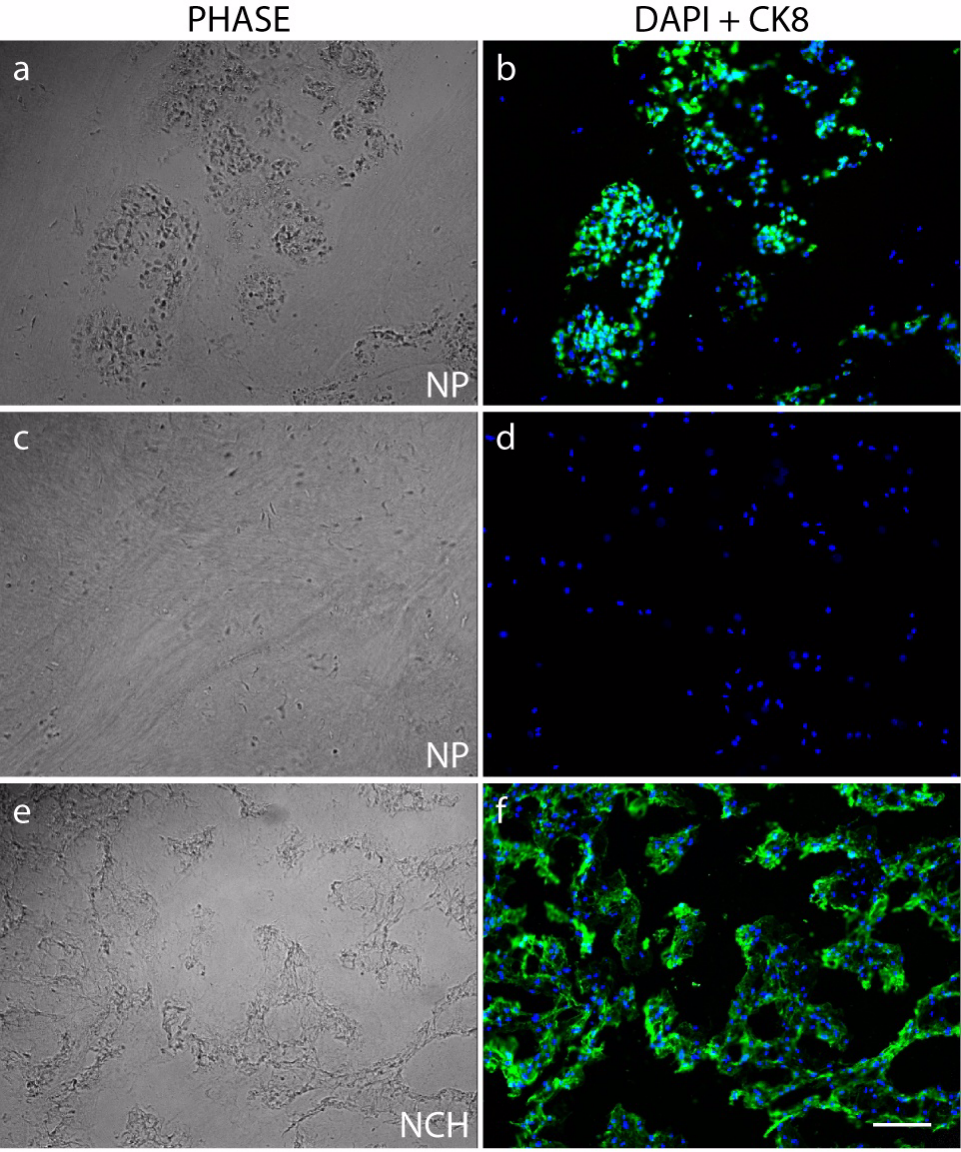
**Immunolocalization of cytokeratin 8 (CK8) in tissue sections**. Immunofluorescence staining was performed on frozen bovine **(a-d) **and porcine **(e,f) **nucleus pulposus (NP) tissue sections. Tissue sections were also imaged under classical phase-contrast microscopy (PHASE), and nuclei were visualized with DAPI (4'-6-diamidino-2-phenylindole) (scale bar = 100 μm). Bovine NP cells were CK8-positive only in cell clusters seen in a gelatinous region of the matrix (a,b). In other areas, no CK8-positive cells were seen (c,d). All cells in porcine notochordal discs (NCH) were CK8-positive (f).

A subset of CK8-immunopositive cells was consistently seen in freshly isolated bovine NP cells. To investigate this expression pattern further, we examined immunofluorescence-labelled tissue sections taken from the NP of the uppermost bovine caudal disc (n = 8 animals). Immunofluorescence of the frozen tissue sections revealed that cells labelled for CK8 were not evenly distributed throughout the tissue but were organized in clusters within a region of matrix which appeared more gelatinous than the surrounding matrix (Figure 3a,b). No fluorescent signal was seen outside these tissue regions (Figure 3c,d). These clusters were found in all animals examined. We also examined CK8 expression in the porcine NP; in these discs, all cells are notochordal; notochordal cells are reported to express cytokeratins [30]. Immunofluorescence of cells from six porcine discs revealed a homogenous population composed entirely of cells expressing CK8 (Figure 3e,f). Negative controls, omitting the primary antibody, were performed in order to ensure that staining did not arise from auto-fluorescence (data not shown). Bovine tissue sections of articular cartilage and AF were also stained for CK8 and did not show any fluorescent signal (data not shown).

### Co-expression of cytokeratin 8 and vimentin by some nucleus pulposus cells

**Figure 4:**
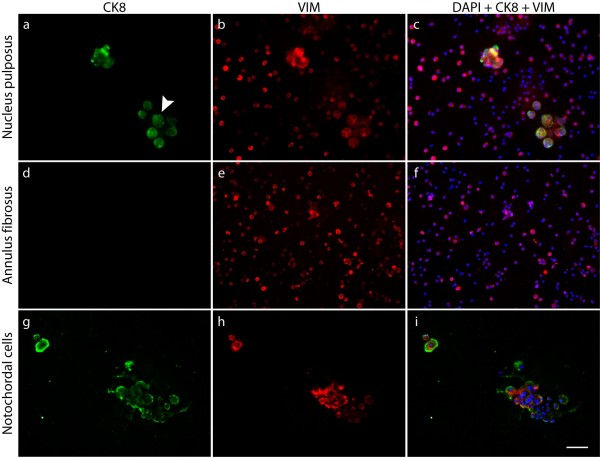
**Cytokeratin 8 (CK8) and vimentin expression in bovine and porcine disc cells**. Dual immunofluorescence staining of CK8 and vimentin (VIM) performed on freshly isolated bovine nucleus pulposus cells **(a-c)**, bovine annulus fibrosus cells **(d-f)**, and porcine notochordal cells **(g-i)**. Nuclei were visualized with DAPI (4'-6-diamidino-2-phenylindole) (scale bar = 60 μm). All cells examined were positive for VIM (b,e,h). No annulus cells were positive for CK8 (d). Some bovine nucleus cells -- forming clusters, as indicated by an arrowhead in (a) -- and all porcine cells (g) were positive for CK8; these cells co-expressed VIM (c,i).

**Figure 5:**
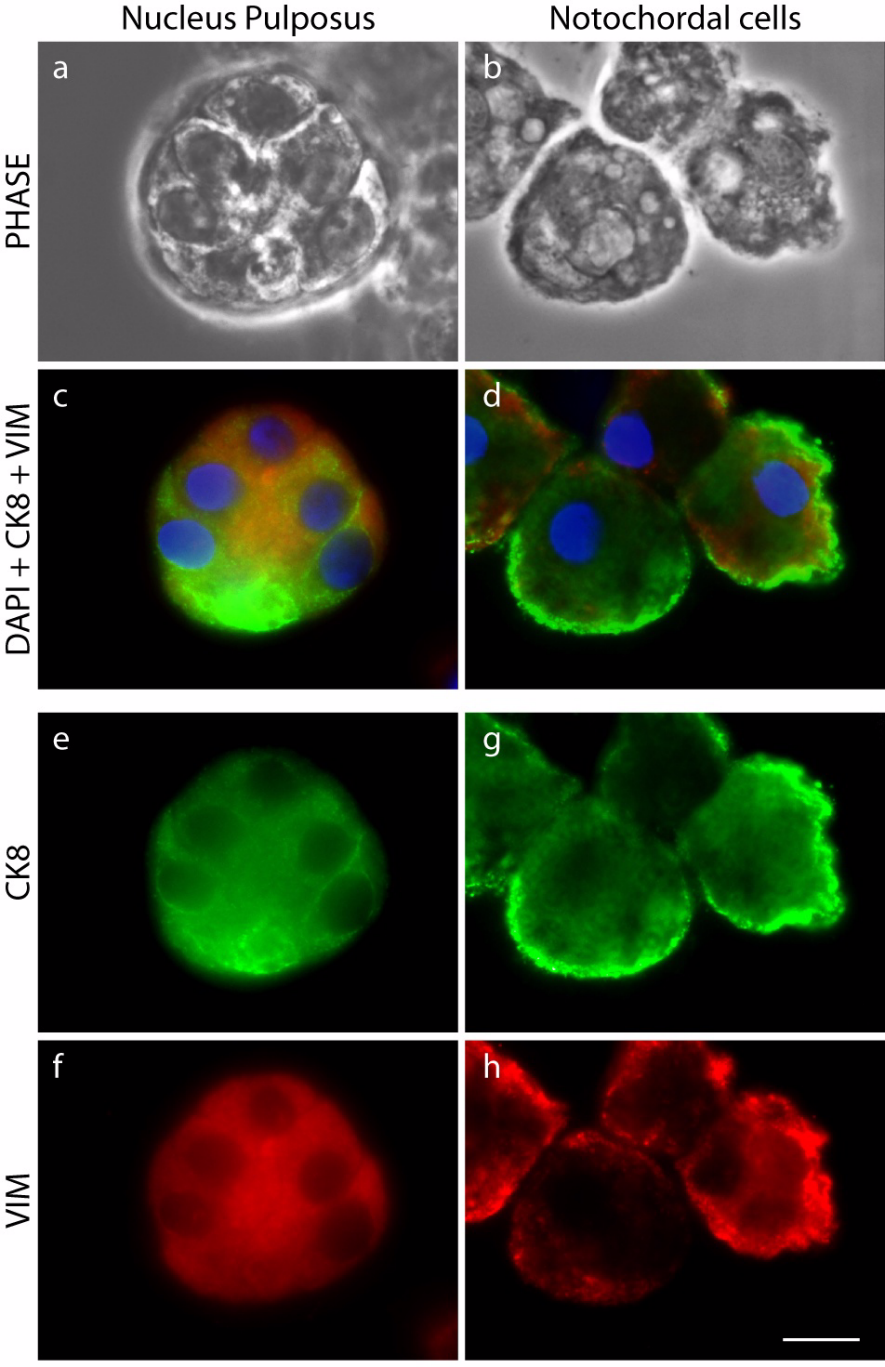
**Cytokeratin 8 (CK8) and vimentin are co-expressed in some bovine nucleus pulposus (NP)-derived cells**. Higher-resolution images showing dual-immunofluorescence staining of CK8 and vimentin (VIM) on freshly isolated NP and notochordal cells. Cells were also imaged under classical phase-contrast microscopy (PHASE), and nuclei were visualized with DAPI (4'-6-diamidino-2-phenylindole) (scale bar = 10 μm). Clusters of bovine NP **(a)**, readily visible as separate cells under PHASE, and of much larger porcine cells **(b) **both co-expressed VIM and CK8 **(c,d)**. However, the organization of these microfilaments appears to differ between the two cell types, being more uniform in the bovine cells **(e,f) **and mainly concentrated toward the plasma membrane in the porcine cells **(g,h)**.

It has been suggested that mature NP cells could originate and migrate from the surrounding cells of the annulus or the cartilaginous end-plates [32] which are mesenchymal in origin. The type III intermediate filament vimentin is used as a marker of cells of mesodermal origin in contrast to the type II to which the epithelial CK8 belongs [33,34]. We therefore examined whether any cells were specific for this type III intermediate filament. Dual-immunofluorescence labelling of freshly isolated cells for CK8 and vimentin was performed in NP and AF from bovine and porcine tissues. The bovine NP cell population, as reported elsewhere [35], all appeared to express vimentin; it was found in both CK8-positive and -negative cells (Figure 4a-c), while the cells of the AF were positive for vimentin only (Figure 4d-f). Co-expression of vimentin and CK8 was consistently restricted to a fraction of the cell population in bovine cells, whereas the porcine notochordal cells stained ubiquitously for both CK8 and vimentin (Figure 4g-i). At higher magnification, the spatial distribution of CK8 and vimentin differed slightly between the two cell types. In bovine NP cells, both intermediate filaments formed extensive networks throughout the cytoplasm (Figure 5c,e,f) while they were mainly compactly organized at the periphery of porcine notochordal cells (Figure 5d,g,h).

### Skeletal maturity and cytokeratin 8 expression in tissue

**Figure 6:**
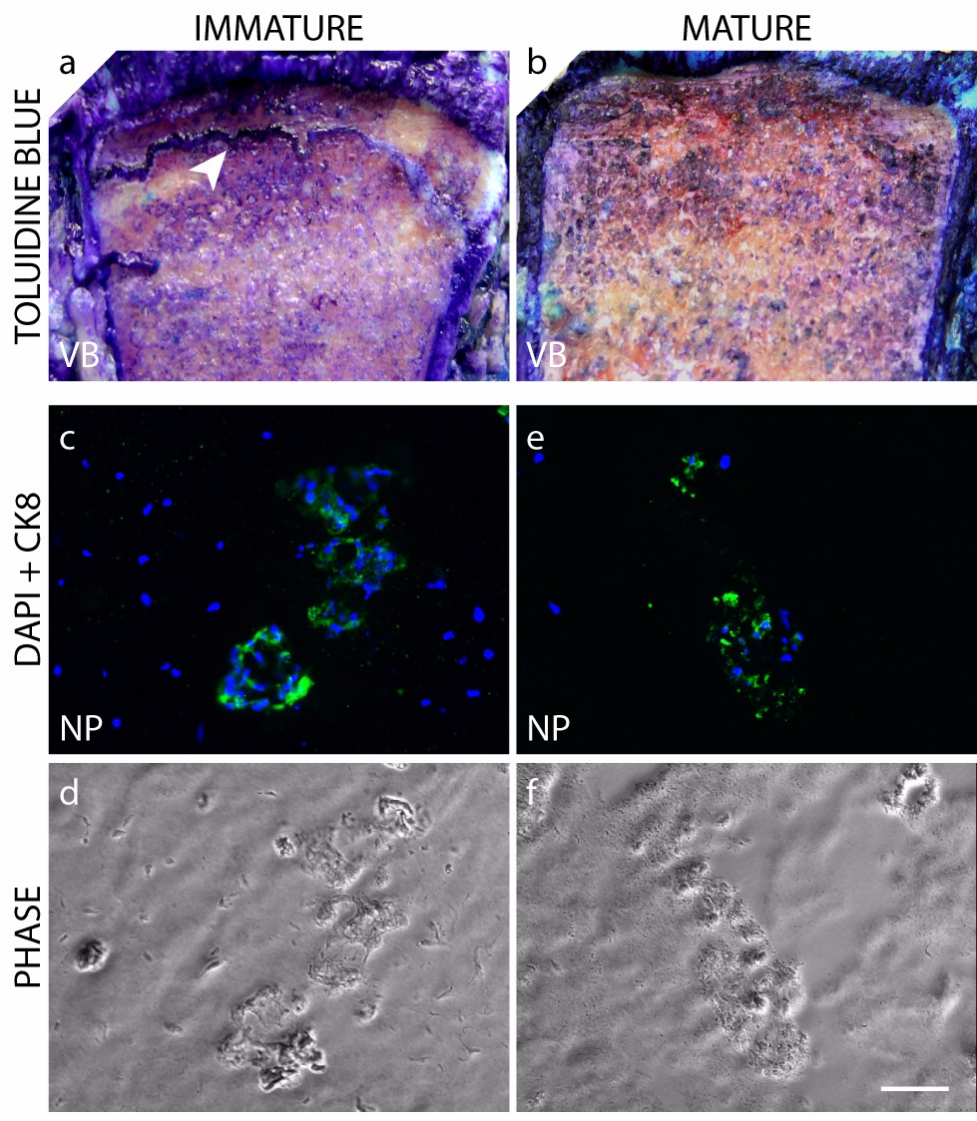
**Presence of cytokeratin 8 (CK8)-positive clusters in the nucleus pulposus (NP) of immature and mature bovine discs**. Toluidine blue staining of the proximal caudal vertebral growth plates (arrowhead) of immature **(a) **and mature **(b) **spinal segments (VB = vertebral body). Immunofluorescence staining of CK8 performed on frozen NP tissue sections taken from the uppermost caudal disc of immature **(c,d) **and mature **(e,f) **bovine tissues. Tissue sections were also imaged under classical phase-contrast microscopy (PHASE), and nuclei were visualized with DAPI (4'-6-diamidino-2-phenylindole) (scale bar = 60 μm).

Since bovine notochordal cells are reported to disappear before birth [22], we investigated the presence of the CK8-positive clusters in the uppermost caudal disc in relation to skeletal maturity. CK8-positive cell clusters were seen, independently of skeletal maturity, in each animal studied (Figure 6c,e).

## Discussion

In this study, we identified two distinct cell populations within the bovine NP based on the expression of the intermediate filament protein CK8. We found by proteomic tools (Figure 1), by immunostaining isolated cells (Figure 2a), and by immunostaining tissue sections (Figure 3) that CK8 was exclusively present in the NP and was not seen in the AF or in articular cartilage. However, we also found that only approximately 10% of the NP cells were positive for CK8 (Figure 2b). These CK8-positive cells were not uniformly distributed throughout the tissue but were grouped in small clusters in isolated regions of the matrix which appeared more gelatinous than the surrounding matrix of the nucleus (Figure 3a,b); these clusters were found, independently of stages of maturity, in all discs examined (Figure 6).

The apparent co-existence of distinct cell populations in the bovine disc raises a number of questions. First, as notochordal disc cells are known to express CK8 [19], are the CK8-positive cells remnants of the original notochordal population of the disc? There are several indications that support this idea. Intermediate filaments such as CK8 are often used to classify the origin of a tissue as differentiated cells usually express only one intermediate filament type; tissues are classified as epithelial when expressing CK8 [36] or as mesenchymal when they express vimentin [33]. Intermediate filaments from two different families can be expressed simultaneously, however, in tumors or in development [37,38]. Disc notochordal cells, epithelial in origin [19], are CK8-positive as expected but are also positive for vimentin [19,30,39] as we saw in the porcine discs (Figures 4 and 5). All bovine NP and also all AF cells were positive for vimentin (Figure 4) as reported previously [35]. Thus, like disc notochordal cells [19,39], the CK8 subpopulation of the bovine NP is also vimentin-positive (Figures 4 and 5), supporting the idea that these cells could have originated from the original population of notochordal cells. This hypothesis is strengthened by the organization of the bovine CK8-positive cells in clusters and by the more gelatinous texture of the surrounding matrix (Figures 3a,b and 6), both features of the notochordal nucleus. However, notochordal cells of the NP have a morphology very different from that of chondrocyte-like nucleus cells as the former are markedly greater in diameter and contain large vacuoles [20,28]; indeed, notochordal cells of the disc are identified mostly by their morphological features [31]. Thus, if the CK8-positive cells are notochordal-like, they would have had to undergo a large size reduction since we found that the size distributions of CK8-positive and -negative cell populations were very similar (Figure 2c). Such loss of size of NP cells has been seen experimentally; the diameter of porcine notochordal cells decreased markedly over 15 days in culture with loss of vacuoles and approached that of chondrocyte-like NP cells [40]. In addition, rabbit notochordal cells have been shown to differentiate toward 'chondrocyte-like' cells when maintained in culture [41]. Thus, on balance, it seems likely that the CK8-positive cells are remnants of the original cell population of the bovine disc. The question, however, could be resolved definitively by gene or proteomic profiling of the two cell populations; at present, only gene profiles from rat notochordal cells and mature chondrodystrophoid canine disc tissue are available [14,15,42].

Second, if, as we suggest above, the bovine disc contains a notochordal-like cell population, does the adult human disc do so too? The NP of bovine discs is reported to be similar to that of human discs in regard both to matrix composition and to cell phenotype [17,22]; thus, if notochordal remnants are present in bovine discs, it is possible that they are also present in adult human discs. If, as in bovine discs, the majority of any remaining CK8-positive cells shrink and lose vacuoles (Figure 2a), only the small fraction of larger cells would be identifiable morphologically as notochordal cells; indeed, some few cells resembling notochordal cells have been reported in adult discs [43,44]. Immunohistochemical studies have, however, identified cytokeratin-positive cells and also the co-expression of CK8 and vimentin in adult human NP [45,46], but whether all cells or only a subpopulation was immunopositive was not stated in these reports.

If, as these various reports suggest, cells from the original notochordal population are retained in discs regarded as non-notochordal, do these cells have any functional significance? Notochordal cell-conditioned medium or co-culture of notochordal and adult NP cells has been found to stimulate matrix production by adult NP cells; in addition, notochordal cells are reported to retard disc degeneration when inserted into damaged rabbit discs [47-50]. These results could be explained by the finding that notochordal cells secrete growth factors that stimulate production of extracellular matrix [21,51]. It has also been suggested that notochordal cell remnants could serve as a stem cell population [20]; indeed, in preliminary experiments, we find that the CK8 population proliferates faster than the CK8-negative population *in vitro *(data not shown). Functionally distinct non-chondrocytic subpopulations have been identified within the NP of adult human discs [52], indicating the possibility that some notochordal cells survive and remain active. Could it be that the presence of these resident notochordal cells and the growth factors they secrete help in the maintenance of a healthy disc, as suggested by Aguiar and colleagues [47] in a study on dog discs, and that these cells are thus essential for disc homeostasis?

Finally, does the presence of CK8-positive cells provide any information on the origin of the chondrocyte-like cells of the adult human or bovine disc? Studies in rabbits suggest that notochordal cells die off to be replaced by mesenchymal cells originating in the inner annulus or cartilage end-plate [32]. However, it has also been suggested that notochordal cells could differentiate into the adult disc nucleus cell phenotype [20] since chordomas, which arise from embryonic notochordal remnants [13], show chondrogenic potential and can differentiate into cartilage-type cells expressing collagen II and aggrecan [53]. Perhaps the possible differentiation pathway from notochordal to mature NP discs should be revisited.

## Conclusions

The finding of a subpopulation of notochordal-like cells in young adult bovine NP is in agreement with a few observations on human discs in the literature [45,46] and stresses the importance of understanding the cell phenotypes of the intervertebral disc. The bovine disc serves as a model of human disc in many studies [25], and the finding that the nucleus contains at least two subpopulations should be considered in experimental design and analysis. In addition, at present, there are many studies investigating the use of MSCs for disc repair [9,54,55]; it is thus essential to understand whether these cells are progenitors of the adult disc population or not [21,31]. In addition, if, as has been suggested [20,31], a subpopulation of notochordal cells is required to maintain disc health, strategies for disc cell repair therapies will need to encompass this cell type.

## Abbreviations

2D: two-dimensional; AC: articular chondrocyte; AF: annulus fibrosus; CK8: cytokeratin 8; DIGE: differential in-gel electrophoresis; DMEM: Dulbecco's modified Eagle's medium; IEF: isoelectric focusing; IPG: immobilized pH gradient; MALDI: matrix-assisted laser desorption/ionization; MSC: mesenchymal stem cell; NP: nucleus pulposus; PBS: phosphate-buffered saline.

## Competing interests

The authors declare that they have no competing interests.

## Authors' contributions

AG designed and carried out the experimental work and drafted the manuscript. MD supervised the DIGE study and carried out the mass spectrometry analysis. JU coordinated the project and revised the manuscript. All authors read and approved the final manuscript.
